# Empirical facts from search for replicable associations between cortical thickness and psychometric variables in healthy adults

**DOI:** 10.1038/s41598-022-17556-7

**Published:** 2022-08-02

**Authors:** Shahrzad Kharabian Masouleh, Simon B. Eickhoff, Somayeh Maleki Balajoo, Eliana Nicolaisen-Sobesky, Bertrand Thirion, Sarah Genon

**Affiliations:** 1 Institute of Neuroscience and Medicine (INM-7: Brain and Behaviour), Research Centre Jülich, Jülich, Germany; 2 Institute of Systems Neuroscience, Heinrich Heine University Düsseldorf, Düsseldorf, Germany; 3 Inria, CEA, Université Paris-Saclay, Palaiseau, France

**Keywords:** Cognitive neuroscience, Standards, Statistical methods

## Abstract

The study of associations between inter-individual differences in brain structure and behaviour has a long history in psychology and neuroscience. Many associations between psychometric data, particularly intelligence and personality measures and local variations of brain structure have been reported. While the impact of such reported associations often goes beyond scientific communities, resonating in the public mind, their replicability is rarely evidenced. Previously, we have shown that associations between psychometric measures and estimates of grey matter volume (GMV) result in rarely replicated findings across large samples of healthy adults. However, the question remains if these observations are at least partly linked to the multidetermined nature of the variations in GMV, particularly within samples with wide age-range. Therefore, here we extended those evaluations and empirically investigated the replicability of associations of a broad range of psychometric variables and cortical thickness in a large cohort of healthy young adults. In line with our observations with GMV, our current analyses revealed low likelihood of significant associations and their rare replication across independent samples. We here discuss the implications of these findings within the context of accumulating evidence of the general poor replicability of structural-brain-behaviour associations, and more broadly of the replication crisis.

## Introduction

One striking fact when studying humans is the obvious inter-individual variability. Inter-individual variability in brain structure at the macroscale can be investigated in individual MRI anatomical scan with different types of measurements. The two most frequently used MR-estimates of grey matter tissue’s features are grey matter volume (GMV) and cortical thickness (CT)^1,2^. Both markers have been used to identify associations between inter-individual variability in brain structure and interindividual variability in behavioral measurements. GMV^3^, as a relatively readily accessible method, has been a measure of choice for popular studies linking inter-individual variability of brain structure to individuals’ skills such as navigation expertise in London taxi drivers^4^ and complex psychological constructs such as political orientation^5^ and social skills (“number of Facebook friends”^6^).

However, in the recent years, questions are raised about the replicability of these associations^7,8^. Subsequently, we performed a systematic and extensive evaluation of the replication rates of SBB-associations using GMV across a range of psychological measurements in a large sample of healthy adults, where we demonstrated that significant associations are very rare and when some are found, they are rarely replicated in an independent sample^9^. While, through detailed evaluations, we demonstrated the influence of multiple factors such as sample size and sample composition on these findings, it could also be argued that the low rate of finding any significant association in the first place and subsequently low replication rate of the significant associations could at least partly be a result of the chosen brain morphometric measure. GMV is frequently seen in the neuroimaging community as an impure, multidetermined, and hence, crude estimate of grey matter tissue^10,11^. Not only none-biological factors, such as misclassification and registration errors, can overshadow interpretations of GMV-variabilities, but also observed inter-individual variations in GMV can be caused by neurobiological changes in structural properties of none-grey matter tissue, such as changes in the underlying white matter, resulting in a change of the folding pattern in the cortical grey matter. Accordingly, it has been shown that variabilities in the cortical volumetric measures are mostly caused by surface area variations and that the cortical volumetric measures show limited sensitivity in detecting slight thickness variations^1^.

CT, on the other hand, is seen as a more straightforward measure of the underlying neuronal features^1,10^ and its variability is expected to show relatively straightforward associations with behavioural measurements. The scientific literature provides a lot of apparent evidence of associations between local thickness and phenotypic and psychometric variables, such as intelligence^12–15^ and personality^16–18^, anxiety trait^19^, impulsivity^20^, musical perception performance^21^ or mentalizing abilities^22^, just to name a few. The availability of cohorts consisting of participants’ genetic information in the recent years, has enabled following up these associations, to identify their genetic underlying factors.

However, the question remains fully open whether the replicability crisis of SBB-studies concerns specifically the search for associations between behavioural variables and GMV, as some may argue, or extend to CT-associations.

In our previous study, we have discussed the issue of spurious correlation as a “by-chance” fitting of noise at the brain structural metrics to noise in the psychometric data. By directly evaluating the replicability of SBB using CT, we aim to discard the hypothesis that noisy structural estimates is specifically related to the method previously used, namely GMV. However, one important point of discussion remains regarding the noisy aspect of psychometric data. In particular, in large collection of healthy adult population data, as used in our previous study, standard psychometric tools are applied to broad population samples and the limited sensibility of these approaches in capturing relevant interindividual differences could be hypothesized. In other words, particularly noisy psychometric data could be assumed as a factor of low replicability in our previous study.

Relatedly, many SBB-studies use composite scores of behaviour, derived across different psychometric variables (for a review, see^23^). These psychometric tools are often validated, for instance by their test–retest reliability^24,25^. Yet use of such composite scores have also led to equivocal conclusions^14,23,26,27^, partly attributed to the diversity of methodological choices (such as the brain atlas used)^26^, or demographic differences^14,26^. However, the extent and the replicability of associations between general composite scores and brain structure, in particular CT has rarely been evaluated.

Hence, in the present study further evaluating the replicability of SBB in healthy adults, we included as behavioural variables, not only specific psychometric variables, but also a composite measure of cognitive abilities derived across different tests in order to evaluate whether association with brain structure can be readily found and whether better replicability rates could be observed with such (supposedly less noisy) behavioural variables. In order to evaluate the replicability of associations between estimates of CT and behavioural measurements, in particular psychometric data, we here performed an extensive empirical evaluation using the high-quality dataset provided by the Human Connectome Project. Several studies have reported associations between brain structural measurements and behavioural variables in this dataset. Our evaluation was extensive at the behavioural level, including psychometric scores that span cognition (such as visual episodic memory, working memory, cognitive flexibility, among others), emotion (such as emotion recognition, anger affect and anger aggression, among others) and personality (such as neuroticism and extraversion). Thus, the range of behavioral measure probed in this study included not only cognitive measures, but also supposedly stable traits, such as personality traits. In addition, we used behavioural composite scores such as cognition crystal component, cognition fluid component, and cognition total component (for a list of psychometric and composite scores included in this study see Table S2). To systematically evaluate the replicability of SBB, in line with our previous work^9^, we used two approaches for all SBB-associations. First, for each behavioural variable, we searched for whole brain associations across 100 random samples of unrelated individuals and examined the spatial consistency of the findings of the CT-associations across the 100 samples. These 100 samples can be considered as 100 different studies (possibly performed by 100 different groups of scientists) that each use a subsample of individuals drawn from the same main cohort to study associations between a certain behavioural variable and CT.

Second, for each behavioural variable we identified the clusters of vertices in the brain that were significantly associated with this variable in one of the previously-mentioned random samples and then investigated whether we could replicate the found association, using a region of interest approach and several different criteria for replication, in an age- and gender-matched subsample. We also further investigated the influence of sample size on replicability. Similar to our previous study using GMV^9^, for all approaches used here, we first provided the pattern of associations found with age, which can be considered as a reliably-measured variable and reasonably expected to correlate with CT. Accordingly, thickness-associations with age, despite the narrow age-range in the HCP, serve here as a benchmark against which the replicability of SBB can be evaluated. Finally, to enable comparison with our previous work and broaden the conclusions to the other commonly-used gray matter morphological property, we also reported associations of the same psychological scores and GMV within the same cohort.

## Results

A total of 10,200 exploratory whole brain SBB-associations (each with 1000 permutations) were tested to empirically identify the replicability of the associations of 34 psychological scores with CT over 100 splits in independent matched subsamples, at three pre-defined sample sizes, within the HCP cohort (see Supplementary Table S1, for the total number of participants with available score for each of the psychological scores).

Altogether, in contrast to CT-associations with age, significant SBB-associations were highly unlikely and only infrequently observed. For the majority of the tested psychological variables no significant association with CT were found in more than 90% of the whole brain analyses.

### Replicability of “whole brain exploratory SBB-associations”

**Figure 1:**
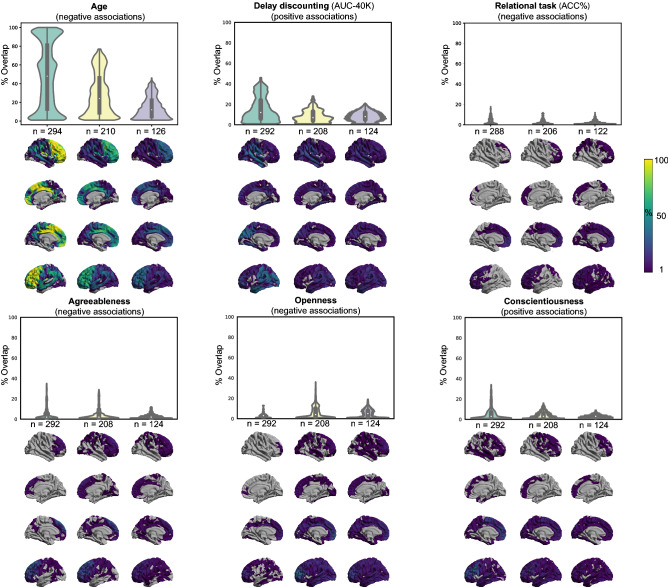
Replicability of exploratory results. Frequency of spatial overlap (density plots and aggregate maps) of significant findings from exploratory analysis over 100 random subsamples, calculated for three different sample sizes (x-axis). Here in addition to age, which is used as a benchmark, the top five behavioral scores with the highest frequency of overlapping findings are depicted. Brighter colors on spatial maps denote higher number of samples with a significant association at the respective vertex. *AUC* area under the curve, *ACC* accuracy.

Ageing associated structural changes: Despite the limited age-range of participants within this sample (28–37 years), in line with previous studies^28^, vertex-wise associations of age with CT were widespread. For most subsamples, we found highly consistent negative associations of CT with age, within the frontal lobe. Aggregate maps of spatial overlap of exploratory findings and density plots, summarizing the distribution of “frequency of significant findings” within each map are shown in Fig. 1A.

When decreasing the sample size of the discovery cohort, the spatial overlap of significant age-associations over 100 splits decreased. More specifically, for the discovery sample of 294 participants, around half of the significant vertices were consistently found as demonstrating significant association between CT and age in 50% of the whole-brain exploratory analyses (i.e. rather high level of spatial consistency of significant findings). As the size of the subsamples decreased, the shape of the distribution also changed, and the median of the density plots fell around 30% and even 10% for samples consisting of 210 and 126 individuals, respectively.

These results highlight the influence of sample size on the replicability (frequency of overlap) of whole-brain significant associations, even for age, a straightforward measure not relying on a specific tool, that shows stable associations with variations in CT.

Structural associations of the psychological scores: In contrast, for most of the psychological scores, only few of the 100 discovery subsamples yielded significant clusters. Table 1 and supplementary Table S2 show the number of splits for which the exploratory whole-brain SBB-analysis resulted in *at least one* significant positively or negatively associated cluster for each score. These results reveal that finding significant SBB-associations using the exploratory approach in healthy individuals is highly *unlikely* for most of the psychological variables. Furthermore, the significant findings were spatially very diverse, that is, spatially overlapping findings were very rare.

**Table 1:** Summary of exploratory findings.

	70% discovery/30% test		50% discovery/50% test		30% discovery/70% test	
	# positively associated clusters (split%)	# negatively associated clusters (split%)	# positively associated clusters (split%)	# negatively associated clusters (split%)	# positively associated clusters (split%)	# negatively associated clusters (split%)
Age (years) n-total = 420	0	586 (100%)	0	534 (87%)	0	322 (57%)
Delay discounting (AUC-40 K) n-total = 418	202 (64%)	0	101 (37%)	0	95 (29%)	0
Relational task (ACC %) n-total = 412	0	27 (19%)	0	18 (13%)	1 (1%)	31 (15%)
Agreeableness n-total = 418	0	55 (35%)	0	64 (32%)	0	24 (16%)
Openness n-total = 418	0	30 (13%)	0	71 (39%)	0	51 (26%)
Conscientiousness n-total = 418	76 (34%)	0	43 (18%)	0	24 (13%)	0

For each discovery sample size, the number of clusters in which cortical thickness is positively or negatively associated with the tested phenotypic or psychological score is reported. The number of splits (out of 100) in which the clusters were detected are noted in parentheses (i.e. % of splits with at least one significant cluster [in the respective direction]).

*AUC* area under the curve, *ACC* accuracy.

We here retained for further analyses the five psychological scores for which the discovery samples most frequently resulted in at least one significantly associated cluster. These three scores were the area under the curve for discounting of $40,000 [Delay discounting (AUC-40 K)], the accuracy percentage during the relational blocks from the in scanner relational task [Relational task (accuracy percentage)], and agreeableness, openness, and conscientiousness scores of the five-factor personality model. For example, for the discovery samples of 292 adults, in 64 out of 100 randomly generated discovery samples, at least one cluster [not necessarily overlapping) showed a significant positive association between area under the curve for discounting of $40 K and CT (Table 1)].

Yet again, in line with our observations for age associations, generally, the probability of finding at least one significant cluster tend to decrease in smaller discovery samples (see Table 1). Likewise, as the discovery sample size decreased, the maximum rate of spatial overlap, as denoted by the height of the density plots, decreased (see Fig. 1B–F). The width of these plots shows that the majority (> 50%) of the significant vertices spatially overlapped only in less than 10% of the discovery samples. In the same line, the variability depicted by the spatial maps highlights that many vertices are found as significant only in one out of 100 analyses.

Similarly, as Supplementary Fig. S1 shows, associations of GMV with age highly overlaps across the 100 splits. The top 5 psychometric scores also followed the same pattern as described above. The associations between area under the curve for discounting of $40 K and GMV in some voxels in the temporal lobes were quite stably depicted across the splits and the overlap decreased with decreased sample size. The remaining scores rarely showed associations with GMV and the found associations were not replicated across subsamples.

The replicability results of whole brain CT-associations for these 5 psychological scores, as well as age within the whole HCP sample (including related individuals) are presented in the Supplementary Fig. S4. For this analysis, the exploratory vertex-wise analyses are assessed using PALM, by defining exchangeability blocks (https://fsl.fmrib.ox.ac.uk/fsl/fslwiki/PALM/ExchangeabilityBlocks) of the permutations that shuffle families as a whole.

This analysis corroborated the findings in the unrelated sample, with quite consistent CT-age-associations. Significant structural correlates of behavioral scores were in general very rare and were mostly not overlapping across the 100 splits. However, they demonstrate the same pattern, namely decrease of spatial overlap and more spread associations as the sample size decreased from ~ 700 individuals to ~ 280 individuals.

These results highlight that finding a significant association between normal variations on psychometric data and vertex-wise measures of CT among healthy individuals is highly unlikely, for most of the tested domains. Furthermore, they underscore the extent of spatial inconsistency and the *poor replicability* of the significant SBB-associations from *exploratory analyses*.

### Confirmatory ROI-based SBB-replicability

**Figure 2:**
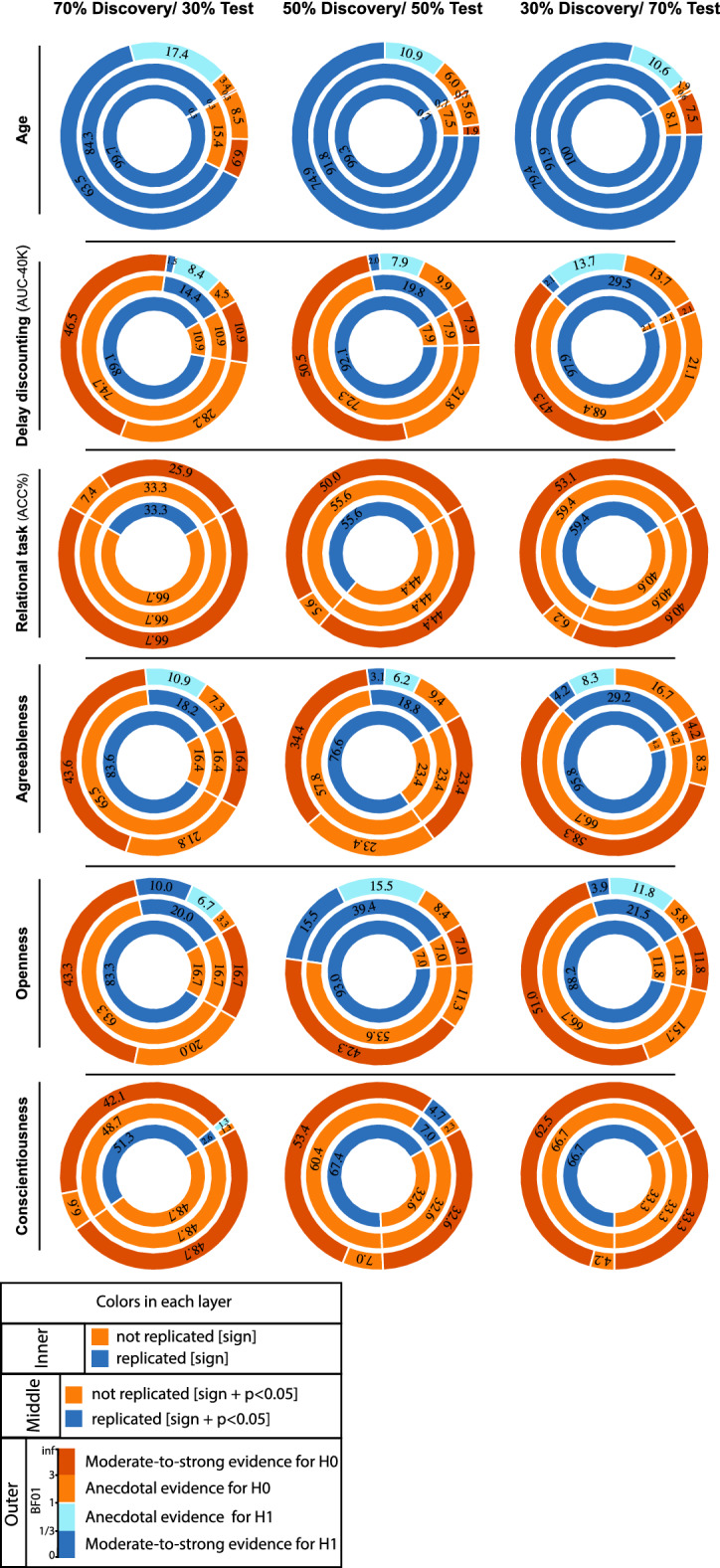
ROI-based confirmatory replication results. Donut plots summerising ROI-based replication rates (% of ROI) using three different critera for three different sample sizes among heathy participants. The most inner layers depict replication using “sign” only (blue: replicated, orange: not replciated). The middle layers define replication based on similar “sign” as well as “statistical significance” (i.e. *p* < 0.05) (blue: replicated, orange: not replciate). The most outer layers define replication using “bayes factor” (blue: “moderate-to-string evidece for H1, light blue: anecdotal evidence for H1; light orange: anecdotal evidence for H0, orange: “moderate-to-string evidece for H0).

Age effects: Over all three tested sample sizes, in more than 99% of the a-priori defined ROIs, age associations were found to be in the same “direction” in the discovery and test samples (i.e., replicated based on “sign” criteria). The examination of replicated findings based on “statistical significance” revealed replicated effects, after Bonferroni correction, in more than 72% of ROIs. This rate of ROI-based replicability increased from ~ 72 to 85%, as the test sample size increased from 126 to 294 individuals (see Fig. 2). Furthermore, as the dark blue segments in the outer layers of Fig. 2 indicate, Bayesian hypothesis testing revealed moderate-to-strong evidence for H1 in more than 63% of the ROIs.

Psychological variables: Fig. 2 also illustrates the replicability rates of structural associations of the top five psychological measures from the whole brain analyses (the area under the curve for discounting of $40 K, accuracy percentage of the relational task, and the three personality scores: agreeableness, openness and conscientiousness).

Despite the mean thickness associations of delay discounting (AUC-40K) being in the same direction in the discovery and test samples (positive SBB-association), for most of the ROIs (> 89%), only less than 9% of all ROIs showed replicated effects based on “statistical significance” criterion. Finally, less than 3% of the ROIs were identified as “successfully replicated” based on the Bayes factors (Fig. 2).

For the three tested samples sizes, associations of the accuracy percentage of the relational task and CT were in the same direction (positive SBB-association) in the discovery and test pairs among beyond 33% of ROIs. Nevertheless, associations within none of these ROIs were defined as successfully replicated using the statistical significance or the Bayes factor criteria (Fig. 2).

Among the three top associated personality scores, negative associations between agreeableness and CT were in the same direction as the discovery effects among more than ~ 77% of ROIs. The significant-replication, after Bonferroni correction, was found among 12% to ~ 20% of all ROIs, for the three tested sample sizes, and Bayes factors defined a successful replication in less than 5% of all ROIs. Negative correlations between openness scores and average CT were depicted in ~ 90% of the ROIs, but significant-replication was found in 12 ~ % to 28% of all ROIs, for the three test sample sizes. Along the same line, successful replication based on the Bayes factors was below 16%. Finally, paired-test samples confirmed direction of association between conscientiousness and CT in less than 52% of ROIs. Significant replication, after Bonferroni correction, was found within not more than 5% of ROIs and similarly less than 5% were defined as successfully replicated in the replication sample using the Bayes factor criteria (Fig. 2).

Extreme low replicabilities are also observed for thickness associations of psychometric scores within the whole HCP cohort, where the discovery and replication pairs are taken from different families (Supplementary Fig. S5).

In general, these results show the span of replicability of structural (thickness) associations from highly replicable age-effects to very poorly replicable psychological associations, within the HCP cohort, consisting of young, healthy adults. They also highlight the influence of the sample size, as well as the criteria that is used to define successful replication on the rate of replicability of SBB-effects in independent samples. Associations between GMV and psychometric scores also in general followed the same pattern, where the highest replication was found for the delay discounting (AUC-40K), with only ~ 40% of ROIs show replicated significantly replicating the associations from the discovery cohort (see Supplementary Fig. S2).

### Effect size in the discovery sample and its link with effect size of the test sample and actual replication

**Figure 3:**
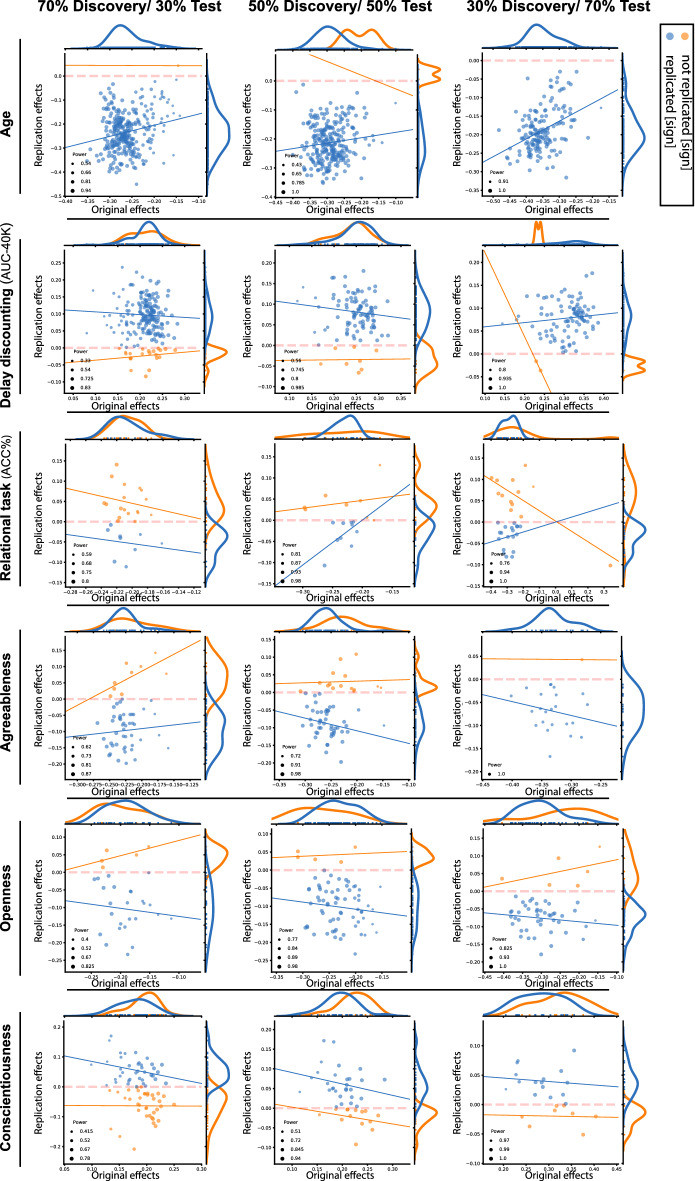
Discovery versus replication effects sizes: Scatter plots of effect sizes in the discovery versus replication sample for all ROIs from 100 splits within healthy cohort; each point denote one ROI, which is color-coded based on its replciation status (by-“sign”). Size of each point is proportional to its estimated statistical power of replication. Regresion lines are drawn for the replciated and unreplicated ROIs, separately.

Figure 3 plots the effect sizes of the discovery versus replication, for the five psychological scores [delay discounting (AUC-40K), relational task (relative accuracy), and the three personality scores: agreeableness, openness, conscientiousness], at three different sample sizes. Overall, the effect sizes were larger in the discovery compared the test cohorts [comparing the marginal distributions on the y- (test sample) and the x-axes (discovery samples)].

Furthermore, focusing on by-“sign” replicated ROIs (blue dots), a positive relationship between the effect sizes of the behavioural associations in the discovery and test samples occurred rarely (blue lines in each subplot).

However, for age-associations we demonstrate a positive correlation between the effect sizes of the discovery and test pairs, showing that the regions with greater negative structural association with age in the discovery sample, also tended to show stronger negative associations within the matched test sample.

Grouping the ROIs into replicated and not-replicated, based on the “statistical significance” criterion, we find a general higher statistical power among the replicated compared to not-replicated ROIs (*p* value of the Mann–Whitney U tests < 10^−2^) for age associations, in contrast to associations with the psychological scores.

See also Supplementary Figs. S4 and S6 for demonstration of relationships between the effect size in the discovery and replication samples, for GMV-associations in the same sample and CT-associations in the whole HCP cohort.

These findings highlight the unreliable aspect of effect size estimations of SBB-associations within the discovery samples among healthy adults, which further result in uninformative estimated statistical power.

## Discussion

Overall our empirical evaluation of the replicability of associations between in-vivo estimates of CT and psychometric variables in healthy adults confirmed our previous findings using GMV^9^. Capitalizing on high-quality neuroimaging data within the HCP cohort, our extensive evaluation across a range of psychometric variables, including composite scores, reveals that significant SBB-associations using in-vivo measurements of CT are very unlikely. When significant associations were found, they were rarely replicated. We here also highlighted the influence of sample size on the replication rates. Hence, overall, the current study extends our previous alarming findings on the poor replicability of SBB-studies to designs using CT estimates and including composite psychometric scores. Below we discuss the implications of these findings, especially in the context of the replicability crisis of psychological, social, and neuroimaging-based neurosciences. Based on this discussion and acknowledging the potential contribution of SBB-associations studies to our understanding of brain-behaviour relationships, we finally propose some possible recommendations for individual studies.

### Unlikely associations between local CT and psychological variables

In this study, we searched for significant associations between CT-variability across the cerebral cortex and inter-individual variability in 34 psychometric variables. For most of the examined psychometric variables (27 out of 34), significant associations were found in less than 10% of our exploratory analyses. Even though the sensitivity of the psychological measures to detect relevant variations in cognition and personality could be questioned, the reliability and relative validity of these tests have been carefully ensured by the HCP initiative^29^. Yet, a relatively low variability due to a potential selection bias of the participants (subsamples from 420 unrelated individuals, and subsamples from the whole HCP-cohort with related individuals) could partly explain the high rates of null findings. This is an important point to consider, as such low variability may not only be a result of a selection bias, but a feature of the commonly used psychometric scores and cognitive tasks, undermining their usefulness in studying neuronal correlates of behaviour^30^.

Nevertheless, we show that, although the age-range is relatively restricted, it shows highly significant replicable associations with CT. Thus, the data here reveals that, although there is inter-individual variability in psychometric data and estimates of CT, significant associations between specific brain regions and specific psychometric variables can hardly be evidenced.

Furthermore, our evaluations also included composite scores of cognition (see Supplementary Fig. S7 for replicability of associations of CT with total cognitive composite score). However, these scores did not show particularly higher replicability rates than individual test scores.

Low consistency of SBB-associations involving composite scores of intelligence and brain structural markers (GMV, among others) have been previously reported^27^, however these inconsistencies have been linked mostly to the use of different sets of psychological measures in deriving the composite scores by the different studies^23^. In the present study, the composite measures were assembled similarly in the discovery and replication samples. We show that, even when assessing replication using a constant study design, reliable associations between scores of general cognitive abilities or general intelligence and CT cannot be evidenced.

In contrast, several scientific publications report significant relationships between interindividual variability in behaviour^19–22^, in particular psychometric intelligence-related^30,31^ and personality-related^17,18^ measures and local thickness. Considering that in the current state-of-the-art, visibility is given mainly to studies which can report a significant finding^31–33^, leaving potentially numerous null findings completely unknown, our in-depth investigations suggests that the current picture of associations between thickness of the cortex and psychometric variables in the scientific and general collective mind could be flawed.

### Spatially inconsistent patterns across the brain with an exploratory approach

In a typical exploratory approach, one or several given psychometric variables of interest are a-priori defined and a mass-univariate analysis is performed. The regions that survive statistical control for multiple testing are then reported. Importantly, traditionally, SBB-studies use low-resolution brain atlases that reflect mainly macro-anatomical boundaries (such as Desikan-Killiany adult cortical atlas^34^, dividing the whole cortex into 68 regions), instead of voxel/vertex-wise data (for a review see^35^). This common approach, in which a broad summarization into macroanatomical territories is imposed to the data, may increase the likelihood of finding irreproducible spatial associations.

Here, we chose a high resolution, vertex-wise approach and by using random splits of a large cohort, we could demonstrate that for a given psychometric variable, the spatial pattern of associations usually varies across the subsamples. Overall, the maximum overlap of significant associations of psychometric measures, across 100 splits, was less than 50% and for most brain regions was much lower. Considering one of the standard personality factors, for example agreeableness, this suggests that if 100 studies, with an identical experimental setting using a mass-univariate approach and within the same population, search for its associations with CT, only five studies would report an association with CT in a given region (e.g. the temporoparietal junction). These findings suggest that inferences about associations between brain structures and behavioural variables should be interpreted with extreme caution.

### Region-of-interest-based replication attempts in paired samples predominantly failed

Estimates of brain structure, such as GMV and CT, are known to vary with demographical variables^28,36^. Accordingly, it could be argued that the poor consistency of the spatial pattern of CT-associations with psychological variables could be due to the demographic diversity across the random splits. Furthermore, multiple comparison corrections, limiting the false positive findings^37^, may increase false negative rates^38,39^ across the exploratory analyses. To address these two issues, we implemented a *confirmatory* approach.

In line with the spatial consistency investigation, this confirmatory approach showed that the significant associations could not be replicated in the majority of the clusters and that the data supported evidence in favour of the null hypothesis (of no association) in the demographically matched samples. Thus, altogether, our study suggests that within a healthy population, significant associations between local CT and psychometric variables are relatively unlikely to be found and when some significant associations are found, they are predominantly not replicable even within an identical experimental setting.

### A replication crisis for structural brain-behaviour studies?

To summarize the current state of the art regarding the replicability of SBB-studies, in 2015, Boekel and collaborators demonstrated that among a few selected structural associations from the previously published studies, most of them could not be replicated. In our previous studies^8,9^ and the current one, we extended the scope of these findings. We showed that, regardless of the neuroimaging measure of brain structure used (be it, GMV or CT) (1) the rate of significant associations between local brain structure and psychometric scores is extremely low; (2) the rate of replication of the found significant associations (after strict statistical threshold), using both exploratory and confirmatory approach is extremely low; (3) replication rate decreases as sample size decreases. Importantly, we had several quality check and associations between age and brain structure served as a benchmark. These control conditions support the validity the statistical approach used in our analyses. It is worth noting as well that our studies showing the low replicability of SBB using voxel-based morphometry and the low replicability of SBB using CT estimates were performed in two different datasets. In other words, the poor replication rates of SBB can hardly be attributed to a questionable quality of the datasets, the chosen morphometric measures or to validity of the behavioural variables. In our previous study, we also demonstrated that the same approach used within a clinical population yielded higher replicability rates suggesting that structural variability (atrophy) and symptomatic behaviour can be reliability related to each other^9^. Altogether these observations raise question about the source of variabilities in commonly-assessed behavioral measures and macroscopic measures of brain structure, such as CT^40,41^, within the healthy populations. We therefore here conclude that the replicability of the standard SBB in healthy populations and hence the validity of this approach in a differential psychology perspective, should be finally questioned. We note, however, that similar issues could be discussed in related fields, such as SBB in psychiatry. Nevertheless, the replicability of structural brain-behavior associations reported in mental disorders have not been empirically addressed and hence should be investigated in future studies.

### Conclusions, recommendations and available resources

The replicability crises that have shaken social^42,43^, psychological^33,43,44^, biomedical and neuroimaging-based neuroscience^37,45–47^ have contributed to the establishment of several recommendations or guidelines, e.g.^45,47–49^. Many of these recommendations are directly relevant in the context of SBB. First, at dataset level, considering the central role of sample size in the replication crisis^9,38^, in line with evidence provided in our study, identification of robust links between psychological variables and brain phenotype needs moving towards big data samples, e.g.^50,51^. Second, because false positives appear to occur frequently and be unnoticed when the degrees of freedom and the exact analyses path leading to the published findings are undisclosed^45,52^, analyses should be documented and disclosure should be increased. Along the same line, the exploratory nature of the analyses should be acknowledged. In particular, considering that the SBB-approach is typically an observational empirical approach, it is frequent that the data has not been acquired for the purpose of the finally reported SBB-analysis. However, if the study was truly a confirmatory study, it should be pre-registered^53,54^. The Open Science Framework (OSF^55^) and many scientific journals offer registration frameworks for empirical research. Furthermore, ideally, the traceability and availability of data and analyses should be ensured. Related to the degree of freedom in the analyses pipelines and computation tools, direct access to the code and data should be provided. SBB-analyses being often based on correlational approaches, they are for example highly susceptible to the effect of outliers. Ideally the reader should be allowed to directly visualize the findings (such as on scatter plots), to evaluate how the results are affected, for example, by a few subjects exclusion. Freely available resources such as knitr (https://yihui.org/knitr/) and datalad (https://www.datalad.org) can be used to promote transparency and traceability and data sharing platforms such as OpenNEURO (https://openneuro.org) and Neurovault^56^ offer free access to data and results of statistical maps. Finally, for any found significant association, replication studies in an independent dataset should be performed. We note however, that the concretization of this suggested good research practice is highly dependent on the active contributions of publishers and funding organizations by providing incentives for replications effort. While these practices could be seen as time consuming and resource consuming in the short-term, they will all contribute to a better understanding of brain-behaviour relationships in the future, but also more generally contribute to build a more optimistic, fruitful, and ethical scientific culture in the long run.

## Materials and methods

### Participants

Healthy adults’ data were selected from the publicly available dataset from the Human Connectome Project (HCP; http://www.humanconnectome.org/). The data were acquired using protocols approved by the Washington University institutional review board. Informed consent was obtained from subjects and consent to publish was obtained from the Human Connectome Project according to the declaration of Helsinki. In addition, the use of the HCP-data was approved by the local ethics committee of the university of Düsseldorf, Germany.

The HCP data comprised from 1206 individuals (656 females), including 298 MZ twins, 188 DZ twins, and 720 singletons, with mean age 28.8 years (SD = 3.7, range 22–37). After passing the HCP quality control and assurance standards^57^, structural data of 1113 individuals were released. The full set of inclusion and exclusion criteria are described elsewhere^50,58^. To avoid overestimating covariance between psychometric and brain structural data due to family relationships between the participants, we selected a subset of unrelated individuals from this cohort, consisting of 420 individuals (age: 28 ± 3.7, 210 females), with good quality structural scans. While the decision to select only unrelated individuals results in lower sample size, it ensures independence of the subjects and thus prevents biases in terms of rate of significant findings and replication. For completeness, however, in the Supplementary material we demonstrate results of the same analyses when the sample is not restricted to unrelated individuals.

### Phenotypical measurements

#### Non-psychological measurements

In line with our previous study^9^, we here used age-associations with the estimates of the CT as a benchmark against which we compare the replicability of behavioural associations.

#### Psychological measurements

The psychological measurements consisted of a subset of 34 standard psychometrics and neuropsychological tests, available in the HCP cohort. The testing consisted of the following main categories:

#### Emotion

 Emotion recognition from the Penn emotion recognition test battery^59^. Negative affect (anger), psychological well-being (life satisfaction) and self-reported measure of emotional support acquired using the NIH toolbox surveys.

#### Cognition

 Card sorting test from the NIH toolbox, measuring executive cognitive flexibility. Area under the curve for discounting of $200 and $40,000^60,61^, as summary measures assessing self-regulation and impulsive behaviour. Reaction time and total number of correct responses from the Penn word memory test, measuring verbal episodic memory. List sorting from NIH toolbox, measuring working memory. Number of correct responses in Penn progressive matrices, measuring fluid intelligence.

Also, few composite cognitive scores were created by averaging various cognitive tests^62^: Crystallized composite score, which is assessed by averaging the normalized scores of each of the NIH Toolbox tests that are crystallized measures (Picture Vocabulary and Reading Tests), measuring verbal reasoning. Early childhood composite cognitive score, which is assessed by averaging the normalized scores of the cognitive measures that comprise the Early Childhood Battery (Picture Vocabulary, Flanker, Dimensional change card sorting and Picture Sequence Memory). The early childhood composite score provides a reliable overall snapshot of general cognitive functioning. Finally, by averaging the normalized scores of each of the fluid and crystallized cognition measures, a total composite score of cognition is generated, measuring levels of cognitive functioning.

In addition to above-mentioned measures, performance (average accuracies and median reaction times) of few in-scanner tasks acquired during functional MRI sessions, were used as additional cognitive scores, consisting of working memory task (two-back working memory tasks for faces, body, tools and places), language task (math) as well as average accuracy of the relational processing task^63^.

Personality: Personality was assessed using five factor personality model of personality^59^.

Furthermore, handedness and dexterity were added as basic motor functions measurements. For all above-mentioned scores we used the unadjusted scores and added age, gender and education as confounders. Supplementary Table S1 demonstrates information about the distribution of the selected scores within the sample of 420 HCP individuals.

### MRI acquisition and preprocessing

MR images were acquired using a 3T Siemens Skyra scanner, consisting of two sets of high-resolution T1- and T2-weighted scans (isotropic 700 µm 3D MPRAGE T1-weighted images: TE/TR/TI = 2.14/2400/1000 ms, field of view (FOV) = 224 mm, flip angle = 8°, Bandwidth (BW) = 210 Hz per pixel; T2-weighted images with identical geometry: TE/TR = 565/3200 ms, variable flip angle, BW = 744 Hz per pixel)^58^. Images underwent gradient nonlinearity correction (using acquired B1 bias field) and the two scans of each modality were co-registered and averaged. CT estimates derived using FreeSurfer v5.3-HCP pipeline (https://github.com/Washington-University/HCPpipelines/blob/master/FreeSurfer/FreeSurferPipeline-v5.3.0-HCP.sh), were downloaded from the Amazon Web Services. This pipeline^58^ is optimized for the HCP data and further incorporates T2-weighted images into the FreeSurfer analysis pipeline.

Each individual’s derived surfaces were then registered to the fsaverage surface (fsaverage—163,842 vertices per hemisphere), through a non-linear surface-based inter-subject registration procedure that aligns the cortical folding patterns of each subject to a standard surface (fsaverage) space^64^.

Finally, each individual’s thickness estimates were mapped to the fsaverage surface and were spatially smoothed on the surface using a gaussian kernel of 15 mm (full-width-half-maximum).

For supplementary GMV-associations, we preprocessed the T1-weighted images using the CAT12 toolbox^65^ and smoothed the volumetric gray matter images with an isotropic Gaussian kernel of 8 mm (full-width-half-maximum).

### Statistical analysis

Exploratory SBB-associations are derived using a mass-univariate approach. Here interindividual variability within the brain structural measure, here CT, at each vertex, is fit to variability of the psychological score using a separate linear model, identifying groups of adjacent vertices (a cluster) that support the link between CT and the tested measure.

To assess replicability of these associations, similar exploration could be performed in another cohort and the spatial location of the significant findings could be compared across cohorts (e.g.^66^). Alternatively, replicability is assessed by focusing on the regions showing a significant SBB-association in the initial exploratory analysis, i.e. regions of interest (ROIs). Existence of association between a summary measure of the brain structure, derived within the ROI, and the same psychological score is then assessed in an independent sample (e.g.^7^). This approach reduces the number of performed tests and thus circumvents the need for the extensive correction across all the vertices within a given region, increasing the replication power.

In line with our previous study^9^, here we assessed replicability of associations between each behavioural measure and grey mater structural variability, using both approaches: the whole brain replication approach and the ROI replication approach, which are explained in detail in the following sections.

### Replicability of whole brain exploratory SBB-associations

Whole-brain analyses: From the main cohort (depending on the analysis, either whole HCP participants or only unrelated individuals) 100 random subsamples (“discovery sample”) are drawn. Within each of these discovery samples, SBB-associations, using a vertex-wise exploratory approach, are examined using the general linear model (GLM) as implemented in the “PALM” tool (https://fsl.fmrib.ox.ac.uk/fsl/fslwiki/PALM). Inference was made using threshold-free cluster enhancement (TFCE)^67^, which unlike other cluster-based thresholding approaches, does not require an arbitrary a-priori cluster forming threshold. *p* Values are determined using 1000 permutations. Further correction due to the number of the hemispheres (as left and right hemispheres are analysed as separate inputs) as well as the two-sided nature of the tests is applied according to^68^. Significant threshold is set at corrected *p* values < 0.05. Age, gender, and education were modelled as confounders. Mean and variance of the respective behavioural score did not differ significantly across all the splits (*p* values of the one-way ANOVA and Levene’s test above 0.95).

Similar to our previous work on GMV^9^, replicability of “whole brain exploratory SBB-associations” across the 100 discovery subsamples, for each psychological score, are demonstrated using spatial consistency maps and density plots. The spatial consistency map denote the frequency of finding a *significant* association between the behavioural score and CT, at each vertex. Accordingly, a vertex with value of 10 in the aggregate surface map has been found to be significantly associated with the phenotypical score in 10 out of 100 discovery samples. Density plots further summarize the spatial consistency maps, demonstrating the distribution of “frequency of significant finding”.

### Replicability of SBB-associations using confirmatory ROI-based approach

Confirmatory analyses: For each “discovery sample”, an age- and gender-matched “test sample” was generated from the remaining participants of the main cohort. For every psychological variable, the significant clusters from the above-mentioned exploratory approach, showing association with the CT in each “discovery sample”, were used as a-priori ROIs. Association between the average CT within each ROI and the psychological variable was assessed using ranked-partial correlation, controlling for confounding factors, and compared between the respective “discovery” and “test” pair subsamples.

Replicability was then quantified according to different indexes (see below) over all ROIs from 100 discovery samples, yielding a percentage of “successfully replicated” surface ROIs based on each index. Here we used same replicability indexes as our previous publication. First, we compare the sign of the ROI-wise correlation coefficients between the discovery and the matched-test samples. Second, statistically significant effects [e.g. after Bonferroni-correction, i.e. *p* value < 0.05/(number of significant clusters from the exploratory analysis of the paired discovery cohort)], in the same direction as the original effects (from the discovery sample), are defined as “successfully” replicated^44^.

Lastly, to compare the evidence that the “test subsample” provided for or against the presence of an association (H1 and H0, respectively), we additionally quantified SBB-replication within each ROI using Bayes factors^69^, which were summarized into four categories (see^9^, for more detail).

### Investigation on factors influencing replicability of SBB-associations

Sample size: To study the influence of sample size on the replicability of SBB-associations, all analyses were performed on three different sample sizes, by generating 100 random splits of the main cohort into age- and gender-matched discovery and test pairs at three pre-defined ratios (70% discovery/30%test; 50% discovery and 50% test; 30% discovery and 70% test).

Effect size: In the confirmatory analyses existence of a positive association between the effect size in the discovery and test pairs is assessed.

Furthermore, we investigated the influence of discovery effect sizes on the rate of finding a “significantly replicated” association in the test subsamples, by comparing the statistical power of replication between the replicated and not-replicated ROIs (here replication was defined using “Statistical Significance” criterion). The replication power was estimated based on the discovery effect sizes and a significant threshold of 0.05 (one-sided) and was calculated using “pwr” library in R (https://www.r-project.org).

## Supplementary Information

Supplementary Information.

## Data Availability

Data, used in this manuscript, is acquired by the Human Connectome Project (HCP). Anonymised data are publicly available from ConnectomeDB (db.humanconnectome.org). Certain parts of the data are available subject to restricted data usage terms. All code used to perform the experiments and prepare figures of this manuscript is available upon Email request: Shahrzad Kharabian Masouleh and will be accessible in the following public repository: https://github.com/shahrzadkh/CTSBB_replicability.
