# Supplementary material for: Empirical facts from search for replicable associations between cortical thickness and psychometric variables in healthy adults
Source: Sci Rep. 2022 Aug 2;12:13286. doi: 10.1038/s41598-022-17556-7 (PMC9345926; doi:10.1038/s41598-022-17556-7)
Supplement: Supplementary file 1 — Supplementary Information. [file 41598_2022_17556_MOESM1_ESM.docx]

*Supplementary Figures:*

**
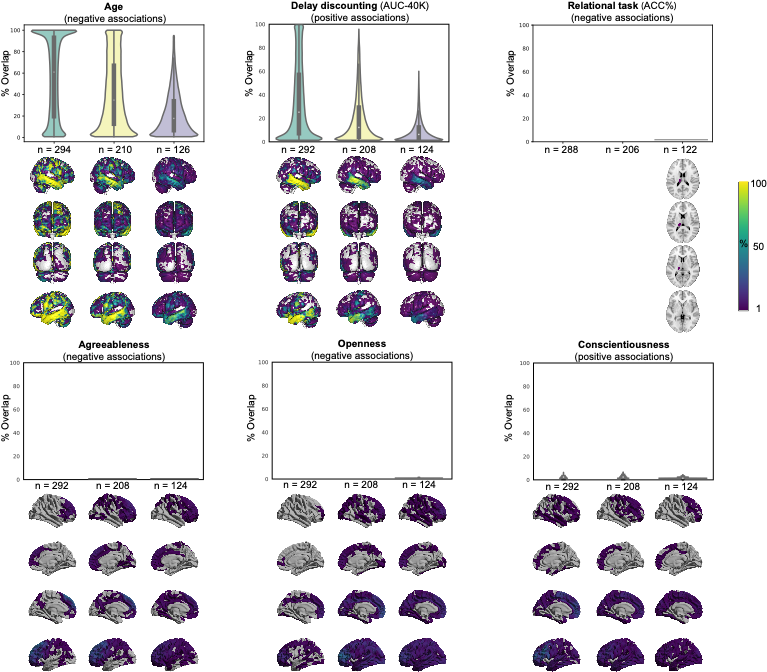
**

***Supplementary Fig. S1. Replicability of exploratory GMV-associations among unrelated individuals in HCP cohort.*** *Frequency of spatial overlap (density plots and aggregate maps) of significant findings from exploratory analysis over 100 random subsamples, calculated for three different sample sizes (x-axis). Brighter colors on spatial maps denote higher number of samples with a significant association at the respective vertex. AUC: Area under the curve; Acc : Accuracy.*

***Supplementary Fig. S2. ROI-based confirmatory replication of GMV associations among unrelated individuals in HCP cohort.*** *Donut plots summarising ROI-based replication rates (% of ROI) using three different criteria for three different sample sizes. The most inner layers depict replication using “sign” only (blue: replicated, orange: not replicated). The middle layers define replication based on similar “sign” as well as “statistical significance” (i.e. p < 0.05 (Bonferroni corrected)) (blue: replicated, orange: not replicate). The most outer layers define replication using “Bayes factor” (blue: “moderate-to-string evidence for H1, light blue: anecdotal evidence for H1; light orange: anecdotal evidence for H0, orange: “moderate-to-string evidence for H0 );*

**
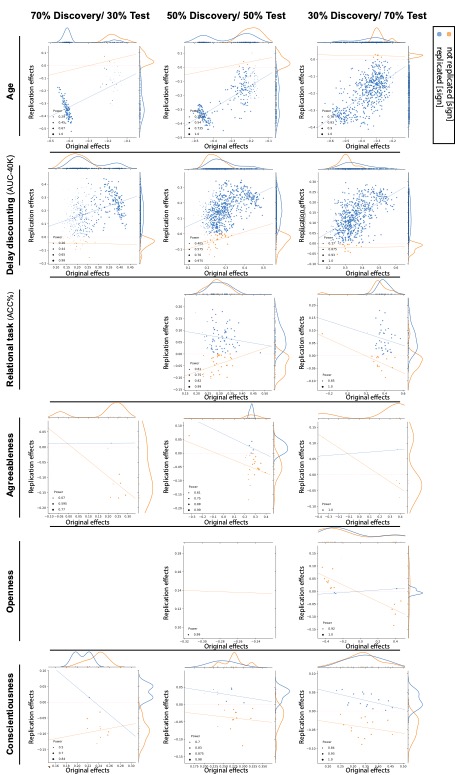
**

**Supplementary Fig. S3. Discovery versus replication effects sizes among unrelated individuals in HCP cohort for GMV associations.** Scatter plots of effect sizes in the discovery versus replication sample for all ROIs from 100 splits; each point denote one ROI, which is color-coded based on its replciation status (by-“sign”). Size of each point is proportional to its estimated statistical power of replication. Regresion lines are drawn for the replciated and unreplicated ROIs, separately.

***
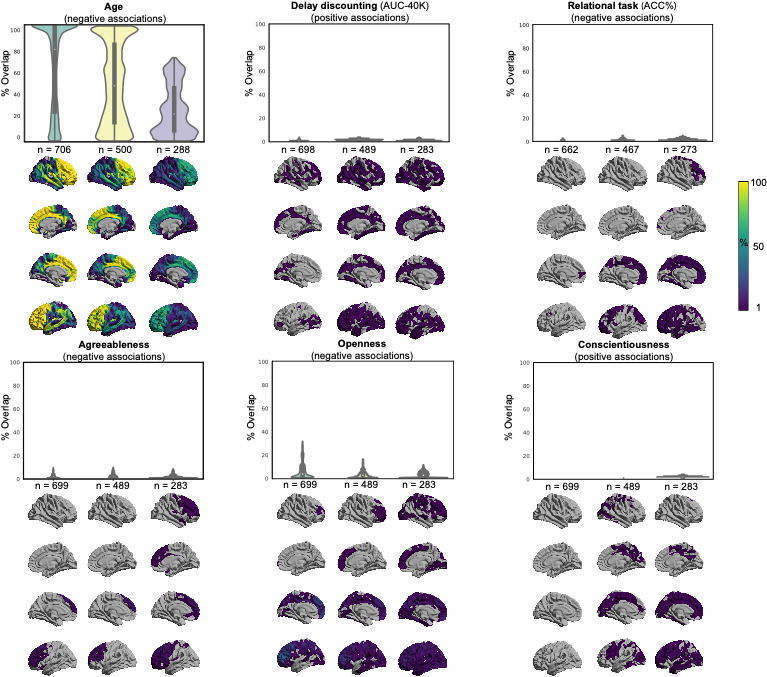
Supplementary Fig. S4. Replicability of exploratory CT-associations in whole HCP cohort.*** *Frequency of spatial overlap (density plots and aggregate maps) of significant findings from exploratory analysis over 100 random subsamples, calculated for three different sample sizes (x-axis). Brighter colors on spatial maps denote higher number of samples with a significant association at the respective vertex. AUC: Area under the curve; Acc : Accuracy.*

******

***Supplementary Fig. S5. ROI-based confirmatory replication of CT-associations in whole HCP cohort.*** *Donut plots summarising ROI-based replication rates (% of ROI) using three different criteria for three different sample sizes. The most inner layers depict replication using “sign” only (blue: replicated, orange: not replicated). The middle layers define replication based on similar “sign” as well as “statistical significance” (i.e. p < 0.05 (Bonferroni corrected)) (blue: replicated, orange: not replicate). The most outer layers define replication using “Bayes factor” (blue: “moderate-to-string evidence for H1, light blue: anecdotal evidence for H1; light orange: anecdotal evidence for H0, orange: “moderate-to-string evidence for H0);*

**
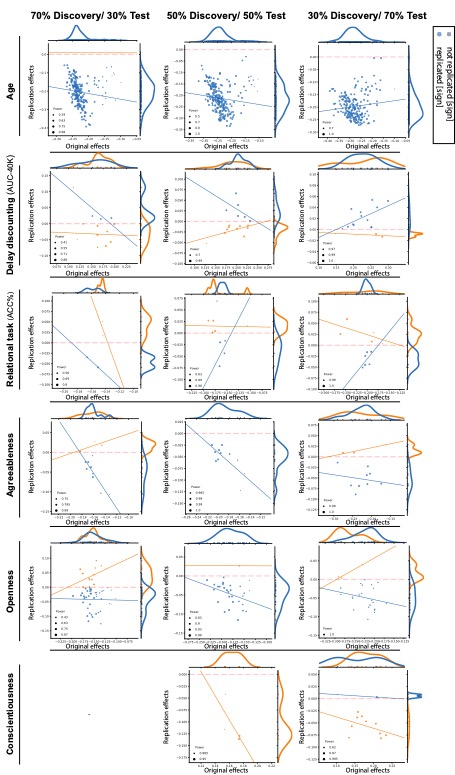
**

**Supplementary Fig. S6. Discovery versus replication effects sizes in whole HCP cohort for CT-associations:** Scatter plots of effect sizes in the discovery versus replication sample for all ROIs from 100 splits; each point denote one ROI, which is color-coded based on its replciation status (by-“sign”). Size of each point is proportional to its estimated statistical power of replication. Regresion lines are drawn for the replciated and unreplicated ROIs, separately.

**
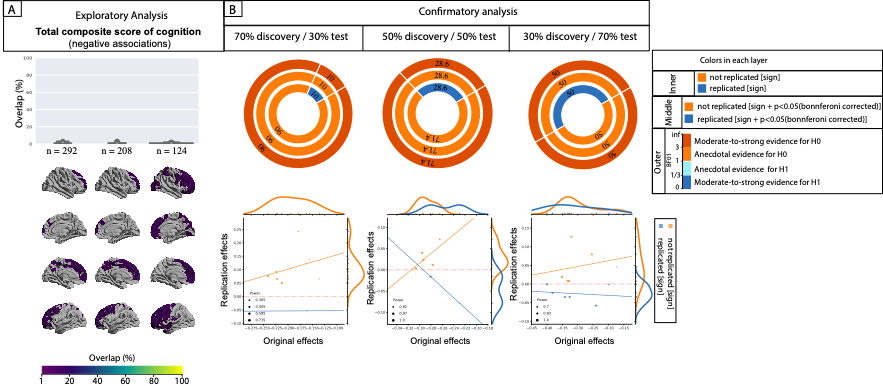
**

***Supplementary Fig. S7. Replication of CT-associations of total composite score of cognition among unrelated individuals in HCP cohort.*** *Frequency of spatial overlap (density plots and aggregate maps) of significant findings from exploratory analysis over 100 random subsamples, calculated for three different sample sizes (x-axis).* *Donut plots summarizing ROI-based replication rates (% of ROI) using three different criteria for three different sample sizes. The most inner layers depict replication using “sign” only (blue: replicated, orange: not replicated). The middle layers define replication based on similar “sign” as well as “statistical significance” (i.e. p < 0.05 (Bonferroni corrected)) (blue: replicated, orange: not replicate). The most outer layers define replication using “Bayes factor” (blue: “moderate-to-string evidence for H1, light blue: anecdotal evidence for H1; light orange: anecdotal evidence for H0, orange: “moderate-to-string evidence for H0);* *Scatter plots of effect sizes in the discovery versus replication sample for all ROIs from 100 splits;* *each point denote one ROI, which is color-coded based on its replciation status (by-“sign”). Size of each point is proportional to its estimated statistical power of replication. Regresion lines are drawn for the replciated and unreplicated ROIs, separately.*

*Supplementary Tables*

**Table S1.** Distribution of raw phenotypical and behavioral scores in the whole sample.

| **Healthy sample** | **Participants** (n = 420 ; 210 male) |
| --- | --- |
| Age (years)  n-total = 420 | 28.1 $\pm$3.7 (22,37) |
| Education (years)  n-total = 420 | 14.9 $\pm$1.76 (11,17) |
| Anger Affect  n-total = 420 | 48.5 $\pm$8.56 (28,85.4) |
| Anger Aggression  n-total = 420 | 51.9 $\pm$8.96 (43.4,83.1) |
| Anger Hostility  n-total = 420 | 51.12 $\pm$8.8 (36.6, 74) |
| Card Sorting  n-total = 420 | 114.86 $\pm$10.81 (85,143.94) |
| Cognition Early childhood Component  n-total = 420 | 116.8 $\pm$11.05 (85.6 , 153.5) |
| Cognition Crystal Component  n-total = 420 | 117.54 $\pm$9.85 (90.95,147.56) |
| Cognition Fluid Component  n-total = 418 | 115.4 $\pm$12.1 (84.5 , 145.2) |
| Cognition Total Component  n-total = 418 | 121.9 $\pm$15.01 (84.5,153.4) |
| Delay discounting (AUC $200)  n-total = 418 | 0.26 $\pm$0.2 (0.016,0.98) |
| Delay discounting (AUC $40K)  n-total = 418 | 0.5 $\pm$0.3 (0.016,0.98) |
| Dexterity  n-total = 420 | 112 $\pm$10.7 (85,1,148.7) |
| Emotional support  n-total = 420 | 51.2 $\pm$9.6 (15.9,62.5) |
| Emotion recognition (#correct responses)  n-total = 418 | 35.6 $\pm$2.5 (24,40) |
| Emotion recognition (correct responses time)  n-total = 418 | 1833.8 $\pm$349.36 (1250,5020) |
| Handedness  n-total = 420 | 65.3 $\pm$42.7 (-100,100) |
| Visual episodic memory (# correct responses)  n-total = 418 | 35.7 $\pm$2.8 (26,40) |
| Visual episodic memory (correct responses time)  n-total = 418 | 1560.73 $\pm$304.5 (1012.5,3265.25) |
| Language task (Math)  n-total = 412 | 83.6 $\pm$10.15 (51.7,100) |
| Life satisfaction  n-total = 420 | 54.5 $\pm$9.2 (24.2,74.6) |
| List sorting  n-total = 420 | 111.98 $\pm$11.5 (80.8,144.5) |
| Agreeableness  n-total = 418 | 33.1 $\pm$5.9 (10,48) |
| Conscientiousness  n-total = 418 | 34.4 $\pm$5.9 (11,48) |
| Extraversion  n-total = 418 | 30.5 $\pm$6.03 (10,47) |
| Neuroticism  n-total = 418 | 17 $\pm$7.84 (0,43) |
| Openness  n-total = 418 | 28.6 $\pm$6.2 (10,45) |
| PMAT (# correct responses)  n-total = 418 | 16.8 $\pm$4.9 (6,24) |
| Relational task (ACC %)  n-total = 412 | 65.18 $\pm$17.64 (16.7,100) |
| WM Task [2back; Body] (ACC %)  n-total = 418 | 76.84 $\pm$14.3 (18.75,100) |
| WM Task [2back; Body] (median reaction time)  n-total = 418 | 1082.69 $\pm$169.9 (676.5,1514) |
| WM Task [2back; Face] (ACC %)  n-total = 418 | 89.5 $\pm$10.3 (50,100) |
| WM Task [2back; Face] (median reaction time)  n-total = 418 | 924.9 $\pm$170.86 (582,1545.25) |
| WM Task [2back; Place] (ACC %)  n-total = 418 | 90 $\pm$10.3 (31.25,100) |
| WM Task [2back; Place] (median reaction time)  n-total = 418 | 931.12 $\pm$168.99 (518.25,1490) |
| WM Task [2back; Tool] (ACC %)  n-total = 418 | 84.39 $\pm$11.3 (50,100) |
| WM Task [2back; Tool] (median reaction time)  n-total = 418 | 931.47 $\pm$169.43 (543.5,1656.5) |

Data are mean ± SD (minimum-maximum).

* median (minimum-maximum).

Abbreviations: AUC: Area under the curve; ACC: Accuracy; PMAT: Penn matrix test; WM: Working memory;

**Table S2. Summary of exploratory findings.** For each discovery sample size, the number of clusters in which cortical thickness is positively or negatively associated with the tested psychological score is reported. The number of splits (out of 100) in which the clusters were detected are noted in parentheses (i.e. % of splits with at least one significant cluster [in the respective direction]).

|  | 70% Discovery / 30% Test | | 50% Discovery / 50% Test | | 30% Discovery / 70% Test | |
| --- | --- | --- | --- | --- | --- | --- |
|  | # positively associated clusters (split%) | # negatively associated clusters (split%) | # positively associated clusters (split%) | # negatively associated clusters (split%) | # positively associated clusters (split%) | # negatively associated clusters (split%) |
| Anger Affect  n-total = 420 | 6 (3%) | 0 | 12 (4%) | 0 | 5 (3%) | 0 |
| Anger Aggression  n-total = 420 | 26 (13%) | 0 | 14 (10%) | 0 | 28 (13%) | 0 |
| Anger Hostility  n-total = 420 | 6 (4%) | 0 | 1 (1%) | 0 | 10 (6%) | 0 |
| Card Sorting  n-total = 420 | 0 | 0 | 6 (4%) | 0 | 6 (1%) | 0 |
| Cognition Early childhood Component  n-total = 420 | 0 | 0 | 5 (1%) | 3 (2%) | 2 (1%) | 6 (2%) |
| Cognition Crystal Component  n-total = 420 | 0 | 46 (25%) | 0 | 28 (17%) | 0 | 14 (9%) |
| Cognition Fluid Component  n-total = 418 | 0 | 0 | 3 (2%) | 4 (2%) | 5 (2%) | 4 (2%) |
| Cognition Total Component  n-total = 418 | 0 | 10 (3%) | 0 | 7 (5%) | 0 | 12 (4%) |
| Delay discounting (AUC $200)  n-total = 418 | 31 (10%) | 0 | 19 (7%) | 0 | 8 (3%) | 0 |
| Dexterity  n-total = 420 | 3 (2%) | 0 | 2 (1%) | 2 (1%) | 9 (4%) | 2 (1%) |
| Emotional support  n-total = 420 | 1 (1%) | 0 | 11 (5%) | 0 | 13 (4%) | 2 (2%) |
| Emotion recognition (#correct responses)  n-total = 418 | 0 | 0 | 2 (1%) | 0 | 2 (1%) | 0 |
| Emotion recognition (correct responses time)  n-total = 418 | 0 | 0 | 6 (3%) | 0 | 2 (1%) | 2 (1%) |
| Handedness  n-total = 420 | 26 (12%) | 0 | 12 (4%) | 0 | 12 (6%) | 0 |
| Visual episodic memory (# correct responses)  n-total = 418 | 0 | 0 | 0 | 4 (2%) | 0 | 6 (2%) |
| Visual episodic memory (correct responses time)  n-total = 418 | 0 | 4 (2%) | 0 | 0 | 0 | 11 (3%) |
| Language task (Math)  n-total = 412 | 0 | 0 | 6 (2%) | 0 | 3 (1%) | 1 (1%) |
| Life satisfaction  n-total = 420 | 1 (1%) | 0 | 0 | 0 | 0 | 0 |
| List sorting  n-total = 420 | 0 | 4 (3%) | 0 | 11 (7%) | 3 (1%) | 22 (11%) |
| Extraversion  n-total = 418 | 1 (1%) | 0 | 5 (3%) | 0 | 4 (2%) | 0 |
| Neuroticism  n-total = 418 | 46 (12%) | 0 | 24 (14%) | 0 | 11 (5%) | 0 |
| PMAT (# correct responses)  n-total = 418 | 5 (3%) | 0 | 5 (2%) | 0 | 4 (2%) | 0 |
| WM Task [2back; Body] (ACC %)  n-total = 418 | 18 (10%) | 0 | 9 (4%) | 0 | 28 (9%) | 0 |
| WM Task [2back; Body] (median reaction time)  n-total = 418 | 0 | 5 (4%) | 0 | 2 (2%) | 1 (1%) | 11 (3%) |
| WM Task [2back; Face] (ACC %)  n-total = 418 | 15 (7%) | 0 | 15 (6%) | 0 | 13 (4%) | 0 |
| WM Task [2back; Face] (median reaction time)  n-total = 418 | 2 (2%) | 0 | 3 (2%) | 0 | 12 (5%) | 0 |
| WM Task [2back; Place] (ACC %)  n-total = 418 | 0 | 0 | 0 | 0 | 4 (1%) | 0 |
| WM Task [2back; Place] (median reaction time)  n-total = 418 | 0 | 2 (1%) | 0 | 5 (3%) | 1 (1%) | 5 (3%) |
| WM Task [2back; Tool] (ACC %)  n-total = 418 | 0 | 0 | 3 (1%) | 0 | 17 (6%) | 2 (1%) |
| WM Task [2back; Tool] (median reaction time)  n-total = 418 | 1 (1%) | 0 | 8 (4%) | 0 | 11 (5%) | 0 |

Abbreviations: AUC: Area under the curve; ACC: Accuracy; PMAT: Penn matrix test; WM: Working memory;
